# The Effect of Chelated Trace Mineral Supplementation in the Form of Proteinates on Broiler Performance Parameters and Mineral Excretion: A Meta-Analysis

**DOI:** 10.3390/ani15213062

**Published:** 2025-10-22

**Authors:** Laurann Byrne, Stephen Ross, Jules Taylor-Pickard, Richard Murphy

**Affiliations:** 1 Alltech Biotechnology Centre, Summerhill Road, A86 X006 Dunboyne, Ireland; 2 Alltech E-CO_2_, Ryhall Road, Stamford PE9 1TZ, UK

**Keywords:** proteinate trace minerals, proteinate, meta-analysis, broiler, mineral excretion, carbon footprint, sustainability

## Abstract

**Simple Summary:**

This work assessed the effect of incorporating chelated trace minerals in the form of proteinates, in place of inorganic trace minerals, on broiler performance parameters such as total feed intake, average daily feed intake, body weight gain, average daily gain, final body weight, feed conversion ratio and mortality. Two separate meta-analyses were carried out: one assessed the effect of replacing inorganics with levels of proteinates ranging from low to equivalent inorganic levels and the second evaluated the effect of replacing inorganics with proteinates at reduced (50–80%) levels only. Evaluation of mineral excretion levels (Cu, Fe, Mn and Zn), indicative of mineral absorption and bioavailability, was also carried out, as this not only impacts bird health and performance but also environmental parameters such as soil health. Sustainability was assessed using a life cycle assessment, focused on the carbon footprint of broiler production, incorporating changes in feed usage, weight gain, mortality rate, and days to slaughter. Results demonstrated that inclusion of chelated trace minerals in specific proteinate forms positively impacted these key parameters, with lower mineral excretion observed for all minerals selected, fewer carcass losses due to mortality, lower feed intake, higher body weight and average daily gain, and lower feed conversion ratio.

**Abstract:**

Comprehensive Meta-Analysis (CMA) software, using data from 64 global studies (288 dietary assessments, 194,356 broilers) evaluated the effects of substituting inorganic trace minerals (ITM) with proteinate trace minerals (PTM) in broiler diets at various inclusion levels. Replacing ITM with PTM at equivalent (100%) or reduced (11–80%) levels improved performance metrics, showing reduced total feed intake (FI) (−6 g/bird), lower average daily feed intake (ADFI) (−0.43 g/bird), higher average daily gain (ADG) (+0.36g), greater body weight gain (BWG) (+4.29 g/bird), higher final body weight (BW) (+7.50 g/bird), improved feed conversion ratio (FCR) (−1.26%), and lower mortality (−10.95%), all significant (*p* < 0.05). Median mineral inclusion reductions of 40% Cu, 59.82% Fe, 41.41% Mn, and 34.67% Zn had no adverse effects, instead enhancing outcomes. Across 17 studies (25,144 broilers, 85 dietary assessments), mineral excretion decreased significantly with PTM versus ITM by 16% Cu, 14% Fe, 21% Mn, and 15% Zn (*p* < 0.001). When PTM replaced ITM at 50–80% inclusion, further benefits were observed, including lower total FI (−7 g/bird), lower ADFI (−1.07 g/bird), higher ADG (+1.67), higher BWG (+2.65 g/bird), lower FCR (−4.50%) and lower mortality (−11.09%) with mineral inclusion reductions of 17% Cu, 42.16% Fe, 42.89% Mn, and 50% Zn. Meta-regression identified significant influences (*p* < 0.05) from study variables such as strain, study duration, and region. Life cycle assessment modelling demonstrated PTM inclusion lowered gross carbon emissions by 3.5% and lower emission intensities per unit live weight of both feed use and overall lifecycle by 4.5% and 4.1%, respectively on diets of high and low soybean meal inclusion. Overall, replacing ITM with PTM in broiler diets can promote production performance of broilers and lower mineral excretion levels while contributing to a lower CFP.

## 1. Introduction

Demand for poultry meat has increased substantially in recent times due to increasing population size and a greater per-capita consumption [1,2]. With modern genetics, birds are closer to their physiological limits, with nutrition, environment and management becoming increasingly important [3,4]. Advances in poultry nutrition have mainly focused on increased efficiency of nutrient use through feed processing, exogenous enzymes, formulating diets based on available P, digestible amino acids and net energy [3,5]. Trace minerals also play a key role in poultry nutrition but are not often a primary focus when formulating diets. Inorganic trace minerals (ITM), usually comprised of oxides, sulfides or chlorides, are still regularly incorporated into diets even though years of scientific evidence have shown minerals bound to specific ligands comprised of amino acids and small peptides, such as proteinate trace minerals (PTM) in certain ligand configurations, have enhanced stability, absorption and bioavailability properties [6,7,8,9,10,11,12]. Not all ligands confer equal stability; for example, the chelation strength of some amino acids is weaker than others due to their configuration and the presence specific amino acid side-chains can confer greater stability. This has been discussed in greater detail in previous publications, with additional discussion on different forms of PTMs that exist, with research highlighting numerous differences between the products that are primarily dependent on the ligand source and manufacturing process [6,13,14,15]. Effective PTM, such as those in certain proteinate forms, can be included in poultry diets at reduced levels to replace their ITM counterparts with equivalent or better effects on health and production performance of birds [16]. This study employed a meta-analysis to systematically combine data from multiple trials to provide evidence-based conclusions to confirm such benefits.

Improvement of feed efficiency to raise birds that require less feed to reach slaughter weight will have both economic and environmental benefits [17,18,19,20,21]. Precision nutrition can alleviate the negative effects of heat stress and contribute to the improvement of animal health, feed efficiency, uniform growth, meat quality, and also the sustainability of production [22,23,24]. Previous studies which used PTM in place of ITM have reported less mineral excretion in poultry manure, thereby contributing to a more sustainable production system and lower environmental impact [7]. Utilizing data obtained over two decades on the benefits of including specific forms of chelated trace minerals in broilers, this study aims to demonstrate the benefits of including proteinate trace minerals in broiler diet formulations. Employing a meta-analysis approach removes any potential bias and results provide a global view of results over an extended time period. In addition to assessing performance benefits, meta-analysis was used in this study to evaluate the effect of incorporating lower levels of PTM (at inclusion levels of 50–80% of ITM) on Cu, Fe, Mn and Zn excretion levels. Primary outcomes to undergo meta-analysis were as follows: Total FI, ADFI, ADG, BWG, Final BW, FCR and mortality. Hypothesized effects direction for ADG, BWG and Final BW was positive—an increase in these parameters was expected with PTM inclusion in place of ITM. A negative effect direction was expected for Total FI, ADFI, FCR and mortality as a decrease in these parameters is indicative of an improvement. A second meta-analysis focused on mineral excretion of Cu, Fe, Zn and Mn.

Improvements in ADG and BWG can shorten the production cycle or yield heavier birds within the same timeframe; reductions in total FI and ADFI can reduce feed costs and improve profitability; and the associated improvement in FCR means birds require less feed to produce each kilogram of meat, maximizing the return on investment. Reduced mortality can increase the number of birds reaching market weight, thereby improving overall flock performance and consistency and reduce costs associated with disease management. This can contribute to higher net margins, improved resource efficiency and better animal welfare. A life cycle assessment (LCA) was also conducted to model the environmental impact of broiler production systems from cradle to farm gate [25]. Employing the meta-analysis results in a scenario system, enabled quantification of the effect of feeding PTM on the carbon footprint of broiler production, estimating the greenhouse gas emissions intensity per unit of product output.

## 2. Materials and Methods

### 2.1. Literature Search and Selection Criteria

**Figure 1:**
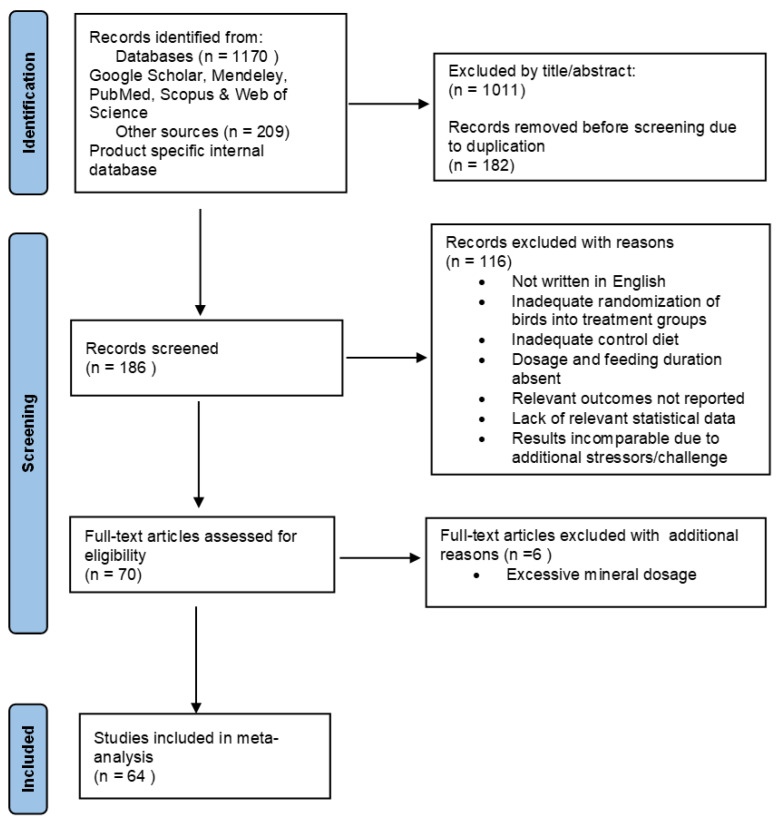
PRISMA flow diagram describing the literature search strategy and study selection for the meta-analysis.

A comprehensive data search was carried out using Google Scholar, Mendeley, PubMed, Web of Science and Scopus databases to obtain peer-reviewed articles evaluating the use of a commercial proteinate trace mineral product (Bioplex Cu, Fe, Mn and Zn, Alltech Inc., Nicholasville, KY, USA) in broiler production. Furthermore, the company’s internal bibliographic database was searched to retrieve published trial reports presented in a Ph.D. dissertation or at international scientific conferences. No unpublished company datasets were used for the meta-analysis. Keywords used for the digital search included “broiler”, “poultry”, “proteinate trace element”, “proteinate trace mineral”, “Bioplex”, “total replacement”, “partial replacement”, “broiler performance” and “mineral excretion”. The literature search and study selection applied in this meta-analysis is reported in Figure 1 according to the Preferred Reporting Items for Systematic Reviews and Meta-Analyses (PRISMA) Statement [25,26,27]. Two reviewers, working independently, screened each record and collected the data. The final literature search to obtain relevant data was conducted in May 2025 and there was no date restriction imposed on the literature search to cover the entire duration that the PTM products have been assessed in broilers.

A total of 186 research articles were initially identified for screening and were subjected to the following criteria: (1) the study was reported in English; (2) the trial was conducted in broilers and adequate randomization of birds into treatments was reported; (3) the study contained at least one negative control (basal diet) and/or positive control (inorganic trace mineral diet), and a diet supplemented with Bioplex^®^ as the proteinate trace mineral product; (4) the trace mineral dosage application rate and feeding duration were reported; (5) information describing the study factors of the experiments were provided or available on request from authors; (6) information of one or more performance parameters and/or mineral excretion was reported or available on request from authors. Post-screening, 64 trials were selected for inclusion in the meta-analysis and are outlined in Table 1.

### 2.2. Data Extraction

Using the data extracted from the 64 selected trials, a database was developed consisting of 288 comparisons of inorganic trace mineral supplemented diets vs. proteinate trace mineral supplemented diets. Although some of the diets compare ITM vs PTM at equivalent inclusion levels, many of the diets compare ITM at 100% inclusion vs. PTM at reduced inclusion levels (11–80%). As such, an additional analysis was carried out to examine the effects of including only lower levels of PTM at 50–80% of ITM.

Trace mineral concentration, form (ITM/PTM) and type (Cu, Fe, Mn and Zn) were recorded for all mineral levels, consisting of both 100% and reduced (11–80%) PTM levels, and reduced (50–80%) levels only (Table 2 and Table 3). Data on production performance parameters (Total FI (kg/bird), ADFI (g/day/bird), ADG (g), BWG (g/bird), FCR (g/feed/g BWG), Final BW (g/bird), mortality (%)) and mineral excretion (Cu (mg/kg), Fe (mg/kg), Mn (mg/kg), Zn (mg/kg)), was also extracted (Table 4). The measure of variance was documented as standard deviation (SD). Additionally, data on various study factors including location, year of study, broiler breed/strain, age of birds, number of birds and study duration was obtained for subsequent meta regression analyses.

### 2.3. Statistical Analysis

Comprehensive Meta-analysis software (version 4, Biostat Inc., Englewood, NJ, USA) was utilized to analyze treatment comparisons in a random-effects model. A random-effect model allowed the true effect to vary from study to study and include between-study variability (true heterogeneity (τ^2^), using the DerSimonian and Laird estimator [88]), as well as sampling error [89]. In instances where a single study reported multiple comparisons that shared a common control group (e.g., different mineral doses or mineral combinations), statistical dependence among effect sizes was addressed by dividing the sample size of the shared control group equally across comparisons. The adjusted data were then analyzed as independent comparisons in accordance with standard meta-analytic practice [89]. For the minimal number of studies with missing standard deviations (SDs), these were excluded if no additional statistics were provided to enable imputation (e.g., sample size and mean). Sensitivity analyses were carried out to ensure results were not significantly affected where this occurred.

Raw mean difference (RMD) at a 95% level of confidence interval (CI) was used to estimate the effect size of dietary PTM supplementation on broiler performance parameters and mineral excretion. In the context of a meta-analysis, the decision to use RMD over SMD depends on the nature of the data, the comparability of the measurement scales and the interpretation of the data that is required. A number of previously published meta-analyses have also worked with RMD or variations thereof and have discussed justifications for such selection [90,91,92,93]. Effect size estimates (RMD) of all trial comparisons on performance variables were declared significant when *p* ≤ 0.05, and a tendency for effect was observed when 0.05 < *p* ≤ 0.10. Furthermore, sub-databases were created to perform meta-regressions of subgroups, evaluating how study factors influenced the response of selected production performance parameters (Total FI, ADFI, ADG, BWG, FCR, Final BW, mortality) and mineral excretion to PTM supplementation. The Knapp–Hartung adjustment was applied to the meta-regression analyses [94]. Study factors consisted of location (Africa, Europe, North America, Oceania, South America, Asia), year of study, broiler breed/strain (Cobb, Cobb 500, Hubbard JV, Ross 308, Ross 708, Arbor Acres, Ross x Cobb, Vencobb), age of birds, number of birds and study duration.

### 2.4. Heterogeneity

Variation across studies was assessed using the *I*^2^ statistic and the associated significance level of chi-squared statistic [89]. *I*^2^ values of <25%, 25 to 50%, and >50% suggest low, moderate, and high heterogeneity, respectively [95].

### 2.5. Publication Bias

Funnel plots were created for each outcome to assess the risk of bias in the studies included in the meta-analysis. The standard error of the observed outcomes as a predictor was used to check for funnel plot asymmetry with symmetrical distribution of studies around the calculated mean difference (MD) indicative of no bias risk. The potential risk of bias was identified by an observed asymmetrical distribution around MD and confirmed by Egger’s test with *p* < 0.05 indicative of the presence of bias in the funnel plot [96]. Egger’s test, which can be unsuitable for low study numbers is fit for purpose in this work as the number of studies assessed for each parameter exceeds 25 (Table 4, Table 5 and Table 6).

### 2.6. Life Cycle Assessment

#### 2.6.1. Scope

**Figure 2:**
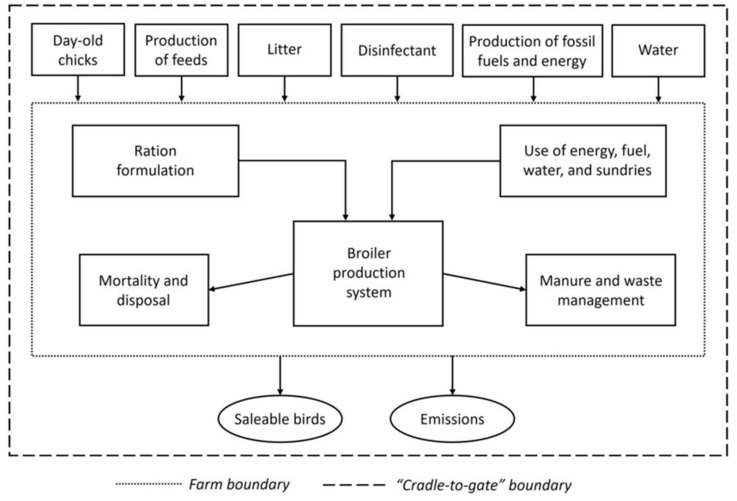
The structure and system boundary of the broiler production system considered in the lifecycle assessment (adapted from Salami et al., 2024 [99]).

Life Cycle Assessment (LCA) simulation modelling was conducted to assess the impact of feeding PTM on the CFP of broiler production. The system boundary encompassed all processes involved in broiler production up to finished birds being ready to leave the farm (i.e., cradle to farmgate). The model therefore accounted for supply chain emissions pre-farm, including the production and delivery of purchased feeds and raw materials, and the emissions from broiler production and litter management on-farm. Downstream emissions attributed to processing, packaging or transport post-farm were not considered, consistent with previous LCA studies of broiler production systems [18,97,98,99]. A simplified flow diagram describing the LCA system boundary is outlined in Figure 2.

The outputs from the broiler system are saleable birds and litter. All litter was exported from the farm and an emissions credit was allocated through a system expansion; therefore, the finished birds were the only true product of the system. The calculated emission intensity was referenced against two functional units: kg CO_2_-eq/bird; and kg CO_2_-eq/kg liveweight (LW). The assessment period was defined as the number of days to reach a finishing weight of 2.5 kg and all scenarios began with a flock of 100,000 birds placed on the farm.

#### 2.6.2. Production System and Scenarios

Production systems modelled in this LCA were based upon an average conventional European housed broiler system. Four scenarios were defined within this production system: a baseline scenario (i.e., without PTM supplementation) and an intervention scenario (i.e., with PTM supplementation) for birds managed on two diets with differing inclusion rates of soybean meal (SBM). Ration formulations were consistent with practical diets employed in a previous LCA study of broiler production [95], defined as low-SBM and high-SBM respectively. Birds in all scenarios were managed on three successive formulated rations according to industry practice. Feed ingredients were consistent in both the baseline and PTM diets other than the PTM supplementation, and all feeds were assumed purchased and delivered onto the broiler farm. Birds were delivered to the farm as day-old chicks from an external hatchery and placed in a flock of 100,000 birds, with the ensuing growing period lasting 44 days in the baseline scenario. All litter was assumed to be exported for use as fertilizer after being stored on-farm until the flock clear-out. All viable finished birds were sold at the production period end, and dead birds were assumed sent to a rendering plant.

#### 2.6.3. Inventory Analysis

Input data on average production system parameters were obtained from 168 commercial European broiler farm environmental assessments of comparable system type conducted by Alltech E-CO_2_ over a 2-year period (2023–2025) (Alltech E-CO_2_, Stamford, UK). Baseline parameters for the broiler cycle were measured as: starting liveweight of day-old chicks (56 g), finished bird liveweight (2.5 kg), mortality (5.2%), daily FI (93.3 g/day), ADG (54.9 g/day), flock period (44 days) and kill out (70.5%). The three broiler rations were defined for starter (day 0–10), grower (day 11–24) and finisher (day 25-finish) phases. The low- and high-SBM diet formulations for each phase are presented in Supplementary Table S1. Dependent variable inputs for the PTM scenario were derived using the observed results from the meta-analysis, applying the relevant percentage change to each production performance parameter. For the PTM scenario these were defined as ADG (56.9 g/day), daily FI (92.3 g/day) and mortality (4.62%). The finished LW of a bird was constant, therefore the observed increase in ADG was projected to result in one fewer day (43 days) on-farm for the flock to reach target LW. Reduced mortality enabled 569 more birds to be finished out of the 100,000 chicks placed, and total feed requirement of the flock was estimated as the product of daily FI × finished birds × days on farm. General resource use on the farm (e.g., bedding, fossil fuels, electricity, disinfectant, and water use), was a fixed variable in the baseline and scenario, obtained from an average of commercial assessment data and based on the starting flock size. Production performance parameters and key LCA input variables for the baseline and PTM scenarios are presented in Supplementary Table S2.

#### 2.6.4. Impact Assessment

Alltech E-CO_2_’s Poultry EA™ (broiler) model (Alltech E-CO_2_, Stamford, UK) was employed to conduct the environmental impact assessment. A sector-specific broiler CFP model, Poultry EA™ is independently accredited to the international product CFP standards PAS:2050 [100] and ISO 14067 [101], and has been employed in assessment of commercial broiler production systems since 2014. Modelling on-farm emissions is consistent with the Intergovernmental Panel on Climate Change (IPCC) guidelines for tier 2 methodology [102], however the tool also has capacity to incorporate case-specific farm data, such as feed intake and dietary crude protein content at a tier 3 level. Nitrogen loss was estimated from the total feed intake per bird, the weighted protein content of the formulated rations, and the estimated percentage of dietary nitrogen excreted by the animal. Direct and indirect emissions of N_2_O and CH_4_ arising from manure and litter management were estimated following the IPCC [96].

The emissions burden associated with the cultivation, processing, and delivery of purchased feeds was estimated using data sourced from FeedPrint (Wageningen University and Research, Wageningen, The Netherlands) [103]. These data were noted to have been derived according to the same specification PAS:2050, and therefore were considered directly compatible with the methodology employed in the present study. Feeds were assumed to be of typical European market production, and SBM was assumed to be of South American origin, including the associated emission burden from land-use change. Emission factors are presented in Supplementary Table S1, estimating the Global Warming Potential (GWP) for each complete formulated ration. Emissions embedded in the production cost of day-old chicks arriving on the farm were obtained from Alltech E-CO_2_’s commercial database, estimated to be 0.35 kg CO_2_-eq per bird. Coefficients for on-farm energy use (electricity, fossil fuels), additional resource use (water, disinfectant, bedding) and any transportation (feed, placed chicks, removal of dead birds) were sourced from the UK Government GHG Conversion Factors for Company Reporting [104]. Exported litter was assumed destined to be used as fertilizer, and accordingly, a carbon offset was applied by the method of system expansion. Nutrient content and availabilities of broiler litter were described by the Agriculture and Horticulture Development Board (AHDB) [105], and coefficients for the avoided production of inorganic fertilizers described by Fertilizers Europe [106]. An assessment of data quality for activity and inventory data is presented in Supplementary Table S3. Data quality assessment employed an approach defined by the Carbon Trust [107], considering the precision, completeness, and temporal, technological and geographic representativeness of the LCA input and inventory data.

Greenhouse gas emissions (kg CO_2_-eq) for each major GHG species were calculated using conversion factors for a 100-year time horizon (GWP_100_), defined to be 28 and 265 times greater than CO_2_ for CH_4_ and N_2_O respectively, as consistent with reporting under the 5th Assessment Report of the IPCC (AR5) [108]. Feed emission intensity (i.e., kgCO_2_-eq attributed to the feed use per functional unit) and total emission intensity (i.e., total kgCO_2_-eq per functional unit) of the production system were subsequently estimated for each of the four scenarios for the production life cycle of 100,000 birds placed in the broiler system.

## 3. Results and Discussion

### 3.1. Study Characteristics

The 64 studies selected for this meta-analysis are outlined in Table 1. Studies were obtained from 19 countries: USA (15), China (8), Australia (7), Brazil (6), Iran (5), The Netherlands (4), India (2), Belgium (2), Slovakia (2), Egypt (2), Turkey (2), Nigeria (2), Pakistan (1), Spain (1), Peru (1), Serbia (1), Thailand (1), Ecuador (1) and Portugal (1) over a 21-year time period (2003–2024). A total of 194,356 broilers were involved in the 288 dietary comparisons comprised of ITM vs. Bioplex PTM supplementation.

Table 2 and Table 3 outline the trace mineral concentrations supplemented in broiler diets and detail the treatment comparisons involving total replacement of ITM with PTM (Bioplex Cu, Fe, Mn and Zn) at all inclusion levels (Table 2) and reduced PTM inclusion levels (Table 3). Results clearly show supplementation levels of PTM were lower for all minerals compared to ITM levels. Many studies have confirmed lower inclusion levels of PTM proteinates are possible due to their enhanced bioavailability and absorption, resulting in a lower requirement without negatively impacting animal health or performance [7,16,32,42,55,82,83,92,109,110,111,112,113,114,115,116,117]. In this work, based on median mineral inclusion levels across all studies, lowered mineral level by 40.00% for Cu, 59.82% for Fe, 41.41% for Mn and 34.67% for Zn resulted in no negative impacts on performance parameters and in fact showed greater performance metric results using PTM in the form of Bioplex proteinates. Similarly, based on median mineral levels comparing reduced PTM levels of 50–80% of ITM, mineral levels lower by 17.00% for Cu, 42.16% for Fe, 42.89% for Mn and 50.00% for Zn were associated with improved performance results (Table 3).

Considerable variation exists in the dataset of production performance parameters and mineral excretion levels included in the meta-analysis (Table 4). A number of factors can contribute to this including, but not limited to: global diversity of studies, mineral supplemental levels varying by region and diet type (starter/finisher), feeding regime, mineral form, maturity stage, breed/strain, stressors/production challenges, length of trial and age of birds. Trial set-up and reporting has evolved over the decades since the first studies were published; however, limitations on data extraction may be encountered in older studies. Based on data availability, a subset of these parameters was selected for the meta-regression analyses.

### 3.2. Production Performance and Mineral Excretion

The results of the overall pooled effect size of production performance parameters and mineral excretion levels when dietary PTM were supplemented in place of ITM at equivalent or lower levels are presented in Table 5. Corresponding forest plots for each variable are presented in Supplementary Figures S1–S11. Lower total FI and ADFI were observed with PTM supplementation (*p* < 0.05) (Table 5, Supplementary Figures S1 and S2). ADG, BWG and final BW were significantly higher (RMD = +0.36, +4.29 and +7.5 g/bird respectively) in PTM supplemented flocks (Table 5 and Supplementary Figures S3–S5). FCR was significantly lower (RMD = −0.02, *p* < 0.05) as was mortality (RMD = −0.73, *p* < 0.05) (Table 5 and Supplementary Figures S6 and S7).

Compared to the ITM control, inclusion of PTM forms of Cu, Fe, Mn and Zn resulted in significantly lower mineral excretion levels (*p* < 0.001): −8.24, −30.01, −43.43 and −27.76 mg/kg respectively (Table 5 and Supplementary Figures S8–S11).

**Table 5:** Overall pooled effects of replacing ITM with PTM (Bioplex Zn, Mn, Cu and Fe) in diets at 11–100% inclusion levels on the productive performance and mineral excretion of broilers trace minerals.

Parameter			Effect Size Estimates			Heterogeneity Tests	
	*N*	Control Mean (SD)	RMD (95% CI)	SE	*p*-Value	*I*^2^ (%)	*p*-Value
**Production performance**							
Total Feed Intake (kg/bird)	190	2.72 (1.90)	−0.006 (−0.011, −0.001)	0.002	0.014	92.106	<0.001
ADFI (g/day/bird)	94	86.99 (40.16)	−0.426 (−0.794, −0.059)	0.187	0.023	98.662	<0.001
ADG (g)	89	48.23 (18.24)	0.358 (0.048, 0.669)	0.158	0.024	98.577	<0.001
BWG (g/bird)	163	1292.01 (878.8)	4.292 (2.821, 5.763)	0.750	<0.001	95.742	<0.001
FCR (g feed/g BWG)	233	1.699 (0.61)	−0.021 (−0.029, −0.009)	0.002	0.001	99.032	<0.001
Final body weight (g/bird)	148	1967.31 (1120.12)	7.501 (1.036, 13.965)	3.298	0.023	99.998	<0.001
Mortality (%)	96	6.64 (8.19)	−0.727 (−1.339, −0.115)	0.312	0.020	99.810	<0.001
**Mineral Excretion**							
Cu (mg/kg)	78	54.85 (23.15)	−8.241 (−9.855, −6.628)	0.823	<0.001	99.678	<0.001
Fe (mg/kg)	76	1198.54 (942.32)	−30.008 (−32.527, −27.489)	1.285	<0.001	98.539	<0.001
Mn (mg/kg)	80	330.00 (175.05)	−43.434 (−45.902, −40.967)	1.259	<0.001	99.790	<0.001
Zn (mg/kg)	76	309.21 (144.89)	−27.759 (−29.367, −26.151)	0.820	<0.001	99.431	<0.001

*N:* number of treatment comparisons; SD: standard deviation; RMD: raw mean difference and its associated 95% confidence interval; SE: standard error. *I*^2^: percentage of variation and associated significance level (*p*-value) of chi-squared statistic.

**Figure 3:**
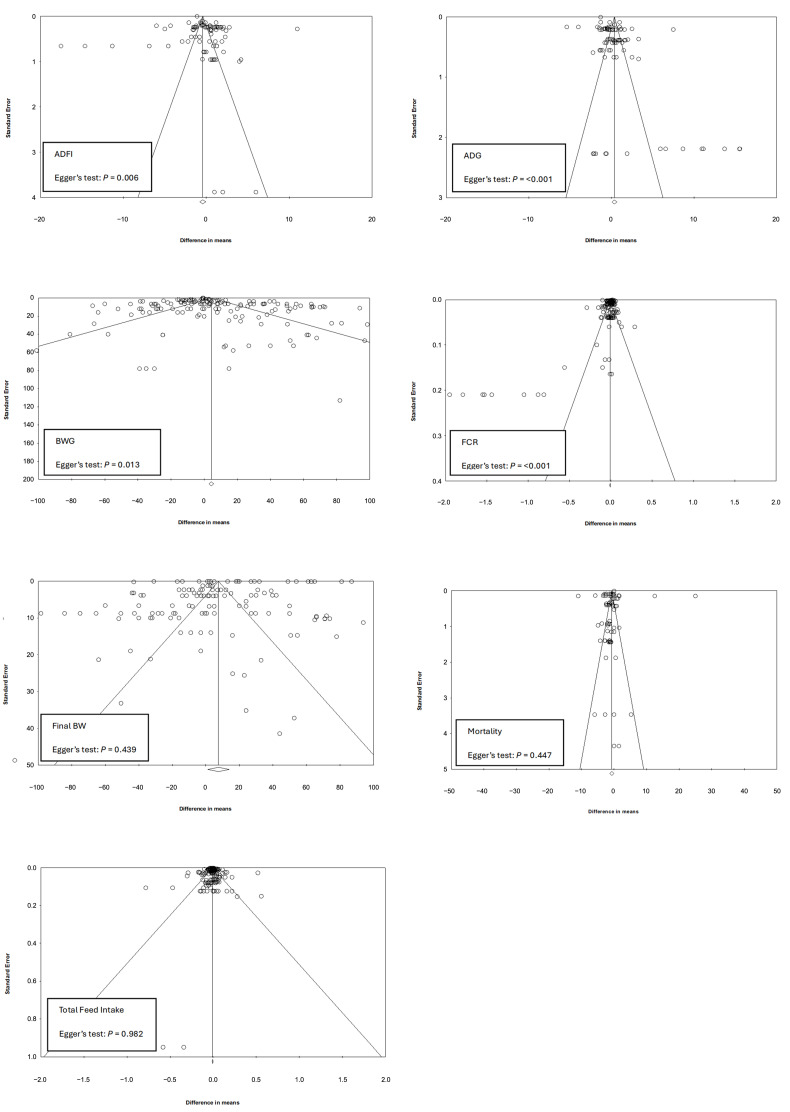
Funnel plots of standard errors by difference in means and the associated significance (*p*-value for Egger’s test) for testing the publication bias of studies included in the meta-analysis for broiler production performance parameters: Total FI, ADFI, ADG, BWG, Final BW, FCR and Mortality. Open circles represent individual study comparisons included in the meta-analysis.

**Figure 4:**
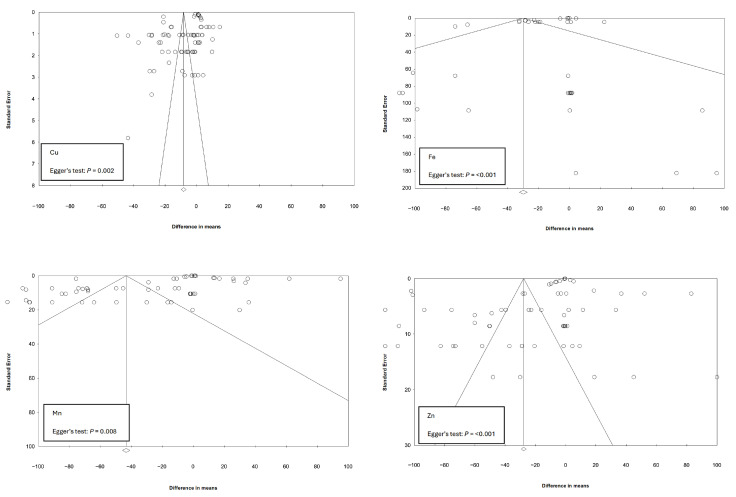
Funnel plots of standard errors by difference in means and the associated significance (*p*-value for Egger’s test) for testing the publication bias of studies included in the meta-analysis for broiler mineral excretion levels Cu, Fe, Mn and Zn. Open circles represent individual study comparisons included in the meta-analysis.

Visual inspection of the funnel plots for all experiments selected for meta-analysis indicates the absence of bias in the included studies due to the symmetrical distribution of the weighted mean difference around standard error (Figure 3 and Figure 4). Additional analyses using Egger’s test returned non-significant values for final BW, mortality and total FI parameters. ADFI, ADG, BWG, FCR and the four mineral excretion datasets returned *p* values of <0.05 indicating possible publication bias; however, the observed asymmetry could be explained by considering meta-regression residuals [118]. Sub-group analysis was carried out to assess this further with the meta-regression indicating effects from a number of study factors. This heterogeneity was expected, considering the studies were performed in different countries under different production management systems.

Table 6 outlines the effect size of production performance parameters when dietary PTM were supplemented at levels of 50–80% of ITM inclusion levels. Results show lower Total FI of 7 g/bird or −0.30% (*p* = 0.054), lower ADFI of 1.07 g/bird or −1.08% (*p* = 0.11), higher ADG of +1.67 g or +3.58% (*p* = 0.001), higher BWG of 2.65 g/bird or +0.21% (*p* < 0.05), lower FCR of 0.076 or −4.50% (*p* = 0.318), and a lower mortality of 0.64 or 11.09% (*p* = 0.50). Although the pooled effect of 50–80% PTM inclusion levels was not significant for FCR (*p* = 0.318), the confidence intervals were narrow (−0.081, −0.023, Table 6) and centered near zero, suggesting the potential effect is likely small rather than absent. For ADFI (*p* = 0.11) and mortality (*p* = 0.50), the effect size magnitude is sizable but the *p*-values were statistically non-significant, likely reflecting context-specific variability. High heterogeneity values (*I*^2^ > 99%, Table 6) for both ADFI and mortality suggest that the true effect varies between studies. The non-significant pooled estimate may simply mean the effects cancel out across differing conditions and not that the intervention has no effect in practice. Comparing the resultant data in Table 6 (reduced 50–80% PTM inclusion levels) with Table 5 (all mineral inclusion levels), further improvements were seen in Total FI (1 g or 0.08%), ADFI (0.64 g or 0.59%), ADG (1.31 g or 2.84%) and mortality (0.09 or 0.14%) with reduced PTM inclusion levels of 50–80%.

**Table 6:** Effect size of replacing ITM with lower inclusion rates (50–80%) PTM (Bioplex Zn, Mn, Cu and Fe) on productive performance parameters of broilers.

Item			Effect Size Estimates			Heterogeneity Tests	
	*N*	Control Mean (SD)	RMD (95% CI)	SE	*p*-Value	*I*^2^ (%)	*p*-Value
**Production performance**							
Total Feed Intake (kg/bird)	51	2.30 (1.69)	−0.007 (−0.015, 0.000)	0.004	0.054	90.256	<0.001
ADFI (g/day/bird)	31	98.77 (43.32)	−1.068 (−2.367, 0.232)	0.663	0.107	99.228	<0.001
ADG (g)	29	46.53 (18.14)	1.666 (0.712, 2.620)	0.487	0.001	98.801	<0.001
BWG (g/bird)	47	1240.97 (875.06)	2.649 (0.276, 5.022)	1.211	0.029	94.280	<0.001
FCR (g feed/g BWG)	71	1.699 (0.96)	−0.076 (−0.081, −0.023)	0.004	0.018	96.527	<0.001
Mortality (%)	25	5.76 (5.29)	−0.639 (−2.513, 1.235)	0.956	0.504	99.632	<0.001

*N:* number of treatment comparisons; SD: standard deviation; RMD: raw mean difference and its associated 95% confidence interval; SE: standard error. *I*^2^: percentage of variation and associated significance level (*p*-value) of chi-squared statistic.

Based on the results obtained for production performance and mineral excretion levels, incorporating PTM, in place of ITM, at either equivalent or lower levels, has a positive impact on the metrics assessed. Previous publications have discussed the benefits of incorporating PTM in poultry nutrition and the findings in this meta-analysis confirm such results [16,112,113,114].

### 3.3. Subgroup Analyses—Location, Year of Study, Breed/Strain, Age of Birds, Number of Birds and Study Duration

Subgroup analysis results are presented in Table 7 and Table 8, showing how various study factors influenced the overall pooled effect size for production performance parameters and mineral excretion levels when PTM replaced ITM at previously stated levels. Table 9 contains the meta-regression results for the lower PTM level (50–80%) inclusion studies. The list of study factors is not exhaustive—other factors such as diet composition, housing, hygiene, genetics and the type of productions system may also contribute. Study factors were selected based on the availability of relevant data in the published studies.

For Total FI at all PTM inclusion levels, certain locations, breeds and the number of birds had an impact (Africa (*p* < 0.05), Asia (*p* < 0.05), Oceania (*p* < 0.001), South America (*p* < 0.05), Ross 308 (*p* < 0.05), Hubbard JV (*p* < 0.05), number of birds (*p* < 0.001)) (Table 7). ADFI at all PTM inclusion levels was significantly impacted by certain locations (Africa (*p* < 0.001)), but other meta-regression parameters assessed were not significant (Table 7). In relation to BWG, many of the study factors had a highly significant (*p* < 0.001) impact including location (Africa and Asia), breed/strain (Arbor Acres, Ross 308 and Vencobb (*p* < 0.05)), age of birds and study duration (Table 7). Final BW was impacted by specific locations (Africa (*p* < 0.001), North America (*p* < 0.001)), certain breeds (Ross 708 (*p* < 0.05)), the age of the birds (*p* < 0.001) and the study duration (*p* < 0.001). Key impacts on FCR (g feed/g BWG) included particular locations (Africa (*p* < 0.001) and North America (*p* < 0.05)) (Table 7). ADG was impacted by certain locations (Africa (*p* < 0.001), Asia (*p* < 0.05)), breeds (Arbor Acres (*p* < 0.05)), the age of the birds (*p* < 0.001), and the study duration (*p* < 0.05) (Table 7). Finally, for mortality, location (Asia (*p* < 0.05)), the age of the birds and the study duration had the most significant impact of the parameters assessed.

Geographical location can influence broiler performance parameters due to differences in environmental conditions, infrastructure and economic factors. As an example, Africa and Asia tend to have greater temperature and humidity extremes compared to other regions. These factors can cause heat stress, which decreases feed intake (lower ADFI), reduces growth rate (lower BWG and ADG) and may increase mortality rates dues to the stress on the birds. In cooler climates with less incidences of heat stress, birds require less energy to regulate body temperature. Certain areas may have higher risks of challenges such as disease outbreaks, which can lead to higher mortality and impact weight gain. Regions with limited access to advanced technology such as automated feeding systems can lead to inconsistent FI and less efficient weight gain. Economic factors such a feed and labour costs and the availability of high-quality feed ingredients may also vary significantly between regions.

Broiler breeds are selected based on specific production characteristics required such as growth rate, feed conversion efficiency or disease resistance. Fast-growing commercial breeds tend to have higher metabolic rates which can cause thermoregulation issues, whereas slower-growing or hybrid breeds may be more resilient under adverse conditions but may have lower BWG metrics [119,120].

Age of birds was selected as a meta-regression parameter for a number of reasons. Feed intake increases with age due to increasing energy and nutrient needs and older birds also tend to have less efficient feed utilisation. Furthermore, older, heavier birds generate more metabolic heat, and are less able to dissipate it, so heat stress effects on ADFI, ADG and mortality tend to increase over time. Younger birds are more vulnerable to environmental stress and disease challenges. Mortality, which is also influenced by genetic selection, is often highest in the first and last weeks [120].

The number of birds can influence results in a number of ways. Firstly, the larger the dataset, the greater the weighting of the result. Secondly, the number of birds in a trial correlates with stocking density. Higher stocking density can reduce individual feed intake per bird due to competition for feeders, restricted movement or stress due to crowding or poor air quality. Elevated density also increases the potential for disease transmission and can lower environmental quality (increased ammonia levels or heat stress) which can directly impact mortality [121,122,123].

In many trials, study duration and age of birds tends to correlate as most trials measured data from day-old chicks to culling. However, a number of studies were based on select time points. Shorter term studies may not capture cumulative or delayed effects such as onset of disease or cumulative stress or feed inefficiencies.

Mineral excretion levels (Cu, Fe, Mn and Zn) were also assessed using a meta-regression of the effects of study factors on the overall pooled effect size in response to PTM supplementation at all levels (Table 8). Most of the parameters selected for inclusion in the meta-regression—location, year of study, breed, age of birds, number of birds and study duration—had a significant (*p* < 0.001) impact on all excreted minerals (Table 8).

The second dataset in this work, consisting only of studies reporting PTM inclusion levels ranging from 80% to 50% of ITM, was also analyzed further using meta-regression (Table 9). For Total FI at reduced PTM inclusion levels, all locations, certain breeds and the number of birds had an impact: Africa (*p* < 0.05), Asia (*p* < 0.05), North America (*p* < 0.05), Oceania (*p* < 0.001), South America (*p* < 0.05), Hubbard JV (*p* < 0.05), number of birds (*p* < 0.001) (Table 9). ADFI at reduced PTM inclusion levels was significantly impacted by certain locations (Africa (*p* < 0.001), but other meta-regression parameters assessed were not significant (Table 9). In relation to BWG, Africa (*p* < 0.001) as a location, Ross 308 and Vencobb (*p* < 0.001) as breeds, the age of the birds (*p* < 0.05), number of birds (*p* < 0.05), and the study duration (*p* < 0.001), were significant factors (Table 9). FCR (g feed/g BWG) was impacted by four of the five locations reported (Africa (*p* < 0.001), Asia (*p* < 0.05), Oceania (*p* < 0.05), and South America (*p* < 0.05)) (Table 9). Parameters that affected ADG included location (Africa (*p* < 0.001)), select breeds (Arbor Acres (*p* < 0.05)), age of birds (*p* < 0.001) and study duration (*p* < 0.001). Mortality was most significantly affected by the age of birds (*p* < 0.05) and the study duration (*p* < 0.05) for studies reporting reduced levels of PTM (Table 9).

Inclusion of a meta-regression ensures additional factors of potential variance are assessed and incorporated into the overall meta-analysis findings, further reducing bias and explaining additional contributing factors of heterogeneity. From the meta-regression results in this study, it is clear that factors assessed had a notable impact on overall results and demonstrate the importance of including extended methods of statistical analysis. However, as previously mentioned in Section 3.1, there are a multitude of additional factors that may also play a role.

### 3.4. Simulated Environmental Impact

The impact of supplementing PTM at 50–80% of ITM levels on the CFP of broiler production was evaluated using gross emissions and the emission intensities of feed and overall production, expressed using two functional units (emissions per bird, emissions per LW), under two diet scenarios (low-SBM and high-SBM). A summary of estimated emissions from contributing categories within the LCA of baseline and PTM scenarios is presented in Supplementary Table S4. Gross emissions decreased by feeding PTM, reducing 16.8 t CO_2_-eq overall in low-SBM and 18.1 t CO_2_-eq in high-SBM. Production and delivery of feeds accounted for an average of 81.6% and 82.6% of total emissions in the low- and high-SBM scenarios respectively. Feed emission intensities were 4.50% lower in the PTM supplemented diets regardless of functional unit for both the low-SBM and high-SBM diet scenarios (−0.18 and −0.20 kg CO_2_-eq/bird, respectively) (Table 10). Total emissions intensities were lower on both diets when supplemented with PTM, by 4.07% (low-SBM) and 4.10% (high-SBM) per unit LW produced (−0.08 and −0.09 kg CO_2_-eq/kg LW, respectively) and per bird finished (−0.21 and −0.22 kg CO_2_-eq/bird). The study results showed that supplementing PTM in place of ITM lowered the CFP of broiler production by an average of −3.5% in low- and high-SBM diets and lowered total emission intensity of production on both diets by an average of −4.1% regardless of functional unit.

Previous LCAs for monogastric species have also noted the appropriateness of the method for assessing the effect of specific feeding practices on the environmental performance of a farm’s product supplied to the market [18,32,95,115]. Performance improvements in ADG, mortality and ADFI resulted not only in greater broiler output with lower emission intensities, but lower gross emissions as well. This dual return is crucial when evaluating the environmental benefit of mitigation options from a life cycle perspective. The extent of real environmental improvement will be sensitive to breed and location as noted in the meta-analysis, in addition to localized farm management practices and the supply chain. As such, the LCA representing a European broiler system serves to demonstrate and reinforce the principle of emissions reduction through improved performance and efficiency. This message is in turn considered applicable to the spectrum of broiler production systems and diets globally. When applied at scale, the cumulative effects of supplementing PTM in place of ITM can contribute to meaningful reductions in the carbon footprint of the poultry industry.

## 4. Conclusions

Trace minerals are required to support development and improve the productive performance of broilers however, the form that the trace mineral is supplemented in diets plays a crucial role [6,13,16,109,112,114]. Mineral proteinates show higher retention rates and relative bioavailability values than inorganic trace minerals; however, differences also arise among proteinate forms depending on the manufacturing process such that not all proteinates are equally effective [13,124]. The data obtained from this set of meta-analyses are generated from specific forms of proteinates (Bioplex Cu, Fe, Mn and Zn, Alltech Inc., Nicholasville, KY, USA) and not applicable to other forms of proteinate trace minerals. Results from this study indicate positive benefits on production performance and mineral excretion levels by incorporating PTM in broiler diets. Furthermore, due to the effective absorption and utilisation of these proteinates, lower levels can be included in broiler diets in place of inorganic trace minerals with no detrimental impact on performance and in fact, improved results are often obtained. When the proteinates were incorporated into broiler diets at either the same or lower levels of PTM the following results were obtained: lower total FI of 6 g/bird, lower ADFI of 0.43 g/bird, higher ADG of +0.36 g, higher BWG of 4.29 g/bird, a higher final BW of 7.50 g/bird, a lower FCR of 0.02 or −1.26%, and lower mortality by 10.95%.

Results from trials replacing ITM with PTM at 50–80% of ITM levels also showed significant benefits in production parameters. Lower total FI by 7 g/bird and ADFI by 1.07 g/bird were observed. Furthermore a +1.67 g higher ADG and a 0.076 or −4.50% lower FCR was also noted. Additionally, improvements were seen with greater BWG of 2.65 g/bird, and less mortality by 11.09%. Significantly lower mineral excretion levels for Cu (−16%), Fe (−14%), Mn (−21%) and Zn (−15%) were evidenced by replacing ITM with PTM in broiler diets.

Sustainability impacts were also assessed focusing on the CFP of broiler production and results indicated lower feed emissions can be achieved by incorporating PTM. Incremental improvements in birds’ performance will resonate across the supply chain, improving the resource use efficiency of feeds and system inputs overall. Furthermore, the simplicity of implementing PTM into a poultry system, and the short flock periods observed in the broiler life cycle compared to other livestock species, makes dietary supplementation a practical and important option to reduce GHG emissions of agricultural production.

## Figures and Tables

**Table 1:** List of studies included in the meta-analysis to evaluate the effect of replacing ITM with PTM (Bioplex Cu, Fe, Mn and Zn) in broiler diets.

Reference	Study Location	Year of Study	Breed/Strain	Age of Birds (d)	Number of Birds per Treatment	PTM Mineral(s) Assessed	Study Duration (wk)
Adegbenjo et al. [28]	Nigeria	2014	Unspecified	112	40	Cu	7.0
Bao et al. [29]	Australia	2007(a)	Cobb ^1^	29	32	Cu, Fe, Mn, Zn	4.1
Aksu et al. [30]	Turkey	2011	Ross 308	42	50	Cu, Mn, Zn	6.0
Abdallah et al. [31]	Egypt	2009	Ross 308	35	150	Cu, Fe, Mn, Zn	5.0
Nollet et al. [32]	Netherlands	2007	Ross 308	39	1020	Cu, Fe, Mn, Zn	5.6
Zhu et al. [33]	China	2019	Ross 308	39	180	Cu, Fe, Mn, Zn	5.6
Vieira et al. [16]	USA	2020	Ross 708	48	208	Cu, Fe, Mn, Zn	6.9
El-Husseiny et al. [34]	Egypt	2012	Arbor Acres	42	72	Cu, Mn, Zn	4.0
Ivanisinova et al. [35]	Slovakia	2016	Ross 308	35	54	Zn	5.0
Lippens et al. [36]	Belgium	2006	Ross 308	42	252	Cu, Fe, Mn, Zn	6.0
Bao et al. [37]	Australia	2007(b)	Cobb ^1^	35	200	Cu, Fe, Mn, Zn	5.0
Ao et al. [38]	USA	2006	Cobb ^1^	21	48	Zn	3.0
Ao and Pierce [39]	USA	2006	Cobb ^1^	21	36	Zn	3.0
Ao et al. [40]	USA	2011	Cobb ^1^	35	264	Cu, Fe, Mn, Zn	5.0
Ao et al. [41]	USA	2009	Cobb ^1^	21	60	Zn	3.0
Ao et al. [42]	USA	2011(b)	Cobb ^1^	42	110	Zn	3.0
Ao et al. (trial 2) [42]	USA	2011(b)	Cobb ^1^	42	160	Zn	6.0
Ao et al. [43]	USA	2017	Cobb ^1^	42	288	Cu, Fe, Mn, Zn	6.0
Ao et al. [44]	USA	2019	Cobb ^1^	28	264	Cu, Fe, Mn, Zn	4.0
Azad et al. [45]	Iran	2020	Ross 308	28	40	Zn	4.0
Bao et al. [46]	Australia	2010(a)	Cobb ^1^	35	48	Cu, Fe, Mn, Zn	5.0
Bao et al. [47]	Australia	2010(b)	Cobb ^1^	35	200	Cu, Fe, Mn, Zn	5.0
Bao et al. [48]	Australia	2009	Cobb ^1^	35	24	Cu, Fe, Mn, Zn	5.0
Baloch et al. [49]	Pakistan	2017	Unspecified	35	80	Cu, Fe, Mn, Zn	5.0
Bortoluzzi et al. [50]	USA	2019	Cobb ^1^	28	64	Zn	4.0
de Carvalho et al. [51]	Brazil	2017	Cobb ^1^	16	50	Cu, Fe, Mn, Zn	1.1
de Carvalho et al. [52]	Brazil	2021	Cobb 500	17	50	Cu, Fe, Mn, Zn	1.3
Das et al. [53]	India	2010	Vencobb	42	40	Cu	6.0
Dudley et al. [54]	USA	2016	Cobb ^1^	21	80	Cu, Fe, Mn, Zn	3.0
Vieira et al. [55]	Brazil	2013	Cobb ^1^	49	250	Cu, Fe, Mn, Zn	7.0
Arnaut et al. [56]	Brazil	2021	Cobb ^1^	17	50	Cu, Fe, Mn, Zn	2.4
Bueno et al. [57]	Brazil	2020	Cobb ^1^	49	128	Cu, Fe, Mn, Zn	7.0
Eivakpour et al. [58]	Iran	2021	Ross 308	45	30	Mn	6.4
da Cruz Ferreira Jnr. et al. [59]	Brazil	2022	Cobb ^1^	17	50	Cu, Fe, Mn, Zn	1.4
Glebocka et al. [60]	Netherlands	2008	Ross 308	39	1020	Cu, Fe, Mn, Zn	5.6
Glebocka et al. (trial 2) [60]	Netherlands	2008	Ross 308	42	252	Cu, Fe, Mn, Zn	6.0
Hu et al. [61]	China	2022	Arbor Acres	39	64	Zn	5.6
IRTA [62]	Spain	2004	Ross 308	35	404	Cu, Fe, Mn, Zn	5.0
Jain et al. [63]	India	2020	Cobb 500	35	18	Zn	5.0
Farhadi Javid et al. [64]	Iran	2020	Ross 308	42	32	Zn	6.0
Jegede et al. [65]	Nigeria	2011	Arbor Acres	56	80	Cu	8.0
Lensing and van der Klis [66]	Netherlands	2006	Ross 308	39	1020	Cu, Fe, Mn, Zn	5.6
Liu et al. [67]	China	2011	Arbor Acres	42	36	Zn	6.0
Liu et al. [68]	China	2012	Arbor Acres	42	64	Cu	6.0
Liu et al. [69]	China	2013	Arbor Acres	21	64	Zn	3.0
Liu et al. [70]	China	2015	Arbor Acres	42	36	Zn	6.0
Ma et al. [71]	China	2014	Arbor Acres	14	64	Fe	2.0
Midilli et al. [72]	Turkey	2014	Ross 308	42	125	Zn	6.0
Mwangi et al. [73]	USA	2017	Cobb 500	21	36	Zn	3.0
Mwangi et al. [74]	USA	2019	Cobb 500	21	36	Zn	3.0
Nollet et al. [75]	Belgium	2008	Ross 308	42	252	Cu, Fe, Mn, Zn	6.0
Nunez et al. [76]	Peru	2022	Cobb ^1^	42	252	Cu, Fe, Mn, Zn	6.0
Peric et al. [77]	Serbia	2007	Hubbard JV	42	600	Cu, Fe, Mn, Zn	6.0
Petrovic et al. [78]	Slovakia	2010	Ross 308	42	50	Cu, Fe, Mn, Zn	6.0
Puangmalee et al. [79]	Thailand	2020	Ross 308	17	200	Cu, Fe, Mn, Zn	2.4
Riboty et al. [80]	Ecuador	2024	Cobb 500	42	132	Zn	6.0
Sahraei et al. [81]	Iran	2014	Ross 308	28	24	Zn	4.0
Samuel et al. [82]	USA	2012	Unspecified	21	80	Cu, Mn, Zn	3.0
Tavares et al. [83]	Portugal	2014	Ross 308	31	59,750	Cu, Mn, Zn	4.4
Zamany et al. [84]	Iran	2023	Arbor Acres	42	156	Cu, Fe, Mn, Zn	6.0
Waldroup et al. [85]	USA	2003	Ross x Cobb	63	400	Cu	9.0
Zhang et al. [86]	China	2016	Arbor Acres	21	90	Fe	3.0
Bao et al. [87]	Australia	2007(c)	Cobb ^1^	35	24	Cu, Fe	5.0
Bao et al. (trial 2) [87]	Australia	2007(c)	Cobb ^1^	35	48	Cu, Fe, Mn, Zn	5.0

PTM: proteinate trace minerals (Bioplex Cu, Fe Mn and Zn); ITM: inorganic trace minerals. ^1^ Specific strain not specified by author.

**Table 2:** Pooled trace mineral concentrations supplemented in broiler diets using ITM or replacement of ITM with PTM (Bioplex Zn, Mn, Cu and Fe) at concentrations ranging from 11 to 100% of ITM levels.

Trace Mineral	ITM Treatments (mg/kg)			PTM Treatments (mg/kg)		
	Median	Minimum	Maximum	Median	Minimum	Maximum
Cu	10	1.5	400.0	6	1.4	400.0
Fe	56	7.5	316.0	22.5	5.5	316.0
Mn	64	12.0	121.0	37.5	7.5	121.0
Zn	60	5.0	200.0	39.2	5.0	200.0

**Table 3:** Pooled trace mineral concentrations supplemented in broiler diets using ITM or replacement of ITM with PTM (Bioplex Zn, Mn, Cu and Fe) at 50–80% of ITM levels.

Trace Mineral	ITM Treatments (mg/kg)			PTM Treatments (mg/kg)		
	Median	Minimum	Maximum	Median	Minimum	Maximum
Cu	10	4	400.0	8.3	4.0	200.0
Fe	60	20	316.0	34.7	10.0	316.0
Mn	90	34.4	121.0	51.4	20.0	121.0
Zn	80	10	200.0	40.0	5.0	150.0

**Table 4:** Descriptive statistics of productive performance and mineral excretion levels included in the meta-analysis.

Item	*N*	Mean	Minimum	Maximum	SD
**Production performance**					
Total Feed Intake (kg/bird)	190	2.71	0.132	7.064	1.90
ADFI (g/day/bird)	94	86.84	15.70	160.00	40.00
ADG (g)	89	48.72	13.50	89.40	18.10
BWG (g/bird)	163	1290.42	59.40	3540.00	888.22
FCR (g feed/g BWG)	233	1.699	1.11	4.73	0.51
Final body weight (g/bird)	148	1971.30	145.81	4353	1126.77
Mortality (%)	96	6.03	0	51.6	8.39
**Mineral Excretion**					
Cu (mg/kg)	78	49.69	4.66	83.24	22.25
Fe (mg/kg)	76	1088.27	35.53	2802.05	889.68
Mn (mg/kg)	80	290.28	17.82	770.00	161.16
Zn (mg/kg)	76	286.36	12.24	554.77	136.69

*N*: number of treatment comparisons; SD: standard deviation.

**Table 7:** Meta-regression of study factors on the overall pooled effect of replacing ITM with PTM (Bioplex Zn, Mn, Cu and Fe) at 11–100% dietary inclusion levels on the productive performance of broilers.

Study Factors	Total Feed Intake (kg/bird)				ADFI (g/day/bird)				BWG (g/bird)			
	Coefficient	SE	*p*-Value	*R*^2^ (%)	Coefficient	SE	*p*-Value	*R*^2^ (%)	Coefficient	SE	*p*-Value	*R*^2^ (%)
**Location**												
Africa	−0.041	0.014	0.004	2.0	−5.429	0.823	<0.001	0.0	66.723	4.432	<0.001	13.0
Asia	−0.031	0.011	0.005		0.815	0.535	0.127		10.170	2.731	<0.001	
North America	−0.010	0.012	0.410		-	-	-		1.636	3.048	0.591	
Oceania	−0.100	0.022	<0.001		-	-	-		−27.857	16.254	0.087	
South America	−0.026	0.011	0.017		0.003	0.684	0.997		−2.917	2.653	0.272	
Europe	Referent ^a^				Referent ^a^				Referent ^a^			
**Year of study**	−0.0003	0.001	0.548	0.0	0.043	0.036	0.229	0.0	−0.247	0.171	0.148	0.0
**Breed/strain**												
Arbor Acres	-	-	-	1.0	−0.021	0.702	0.976	0.0	55.343	12.199	<0.001	1.0
Ross 308	−0.017	0.008	0.027		0.148	0.734	0.840		13.771	2.295	<0.001	
Ross 708	−0.012	0.020	0.533		-	-	-		-	-	-	
Cobb ^b^	0.001	0.007	0.855		0.673	1.134	0.553		−1.414	2.184	0.517	
Vencobb	0.009	0.011	0.405		-	-	-		6.938	2.844	0.015	
Hubbard JV	0.050	0.016	0.002		-	-	-		-	-	-	
Ross x Cobb	-	-	-		-	-	-		-	-	-	
Cobb 500	Referent ^a^				Referent ^a^				Referent ^a^			
**Age of birds**	0.000	<0.001	0.981	0.0	−0.019	0.016	0.255	0.0	0.724	0.071	<0.001	0.0
**Number of birds**	<0.001	0.000	<0.001	0.0	0.000	0.000	0.763	0.0	−0.014	0.008	0.090	0.0
**Study duration**	0.000	<0.001	0.923	0.0	−0.018	0.015	0.249	0.0	0.540	0.059	<0.001	0.0
**Study factors**	**FCR (g feed/g BWG)**				**ADG (g)**				**Mortality (%)**			
	**Coefficient**	**SE**	***p*-value**	***R*^2^ (%)**	**Coefficient**	**SE**	***p*-value**	***R*^2^ (%)**	**Coefficient**	**SE**	***p*-value**	***R*^2^ (%)**
**Location**												
Africa	−0.123	0.014	<0.001	0.0	9.097	0.843	<0.001	0.0	−0.660	1.664	0.692	0.0
Asia	−0.014	0.008	0.070		0.952	0.403	0.018		3.519	1.735	0.043	
North America	−0.021	0.009	0.017		-	-	-		−0.056	1.525	0.971	
Oceania	−0.003	0.016	0.848		-	-	-		0.258	3.214	0.936	
South America	−0.017	0.009	0.065		0.809	0.502	0.107		0.751	1.602	0.639	
Europe	Referent ^a^				Referent ^a^				Referent ^a^			
**Year of study**	−0.001	0.001	0.054	0.0	0.018	0.030	0.562	0.0	−0.032	0.079	0.689	0.0
**Breed/strain**												
Arbor Acres	0.016	0.010	0.103	20.0	1.083	0.501	0.031	0.0	1.364	2.848	0.632	0.0
Ross 308	0.002	0.009	0.810		−0.367	0.531	0.490		−0.878	2.808	0.755	
Ross 708	−0.012	0.016	0.456		-	-	-		−0.869	2.774	0.754	
Cobb ^b^	0.004	0.010	0.698		0.692	0.811	0.394		−0.341	2.792	0.903	
Vencobb	0.008	0.015	0.618		-	-	-		-	-	-	
Hubbard JV	0.011	0.020	0.583		-	-	-		-	-	-	
Ross x Cobb	0.034	0.017	0.050		-	-	-		−0.465	3.676	0.899	
Cobb 500	Referent ^a^				Referent ^a^				Referent ^a^			
**Age of birds**	−0.0002	0.0002	0.364	3.0	0.051	0.014	<0.001	0.0	−0.107	0.030	<0.001	0.0
**Number of birds**	0	0	0.420	0.0	0.000	0.000	0.271	0.0	−0.001	0.003	0.648	0.0
**Study duration**	−0.0002	0.0002	0.388	3.0	0.042	0.013	0.001	0.0	−0.107	0.030	<0.001	0.0
**Study factors**	**Final BW (g/bird)**											
	**Coefficient**	**SE**	***p*-value**	***R*^2^ (%)**								
**Location**												
Africa	80.454	14.806	<0.001	1.0								
Asia	7.670	9.752	0.432									
North America	46.809	10.148	<0.001									
Oceania	-	-	-									
South America	9.941	10.117	0.326									
Europe	Referent ^a^											
**Year of study**	0.558	0.576	0.333	1.0								
**Breed/strain**												
Arbor Acres	73.37	42.802	0.087	1.0								
Ross 308	−9.018	11.861	0.447									
Ross 708	30.084	12.997	0.021									
Cobb ^b^	−7.144	13.704	0.602									
Vencobb	2.539	16.804	0.880									
Hubbard JV	23.716	24.795	0.339									
Ross x Cobb	21.87	22.671	0.335									
Cobb 500	Referent ^a^											
**Age of birds**	0.903	0.209	<0.001	47.0								
**Number of birds**	−0.001	0.001	0.208	1.0								
**Study duration**	0.654	0.179	0.0002	47.0								

SE: standard error; *R*^2^: proportion of between-study variance (heterogeneity) explained by the study factors. ^a^ Study location and broiler breed/strain were estimated by using Europe and Cobb 500 as the baseline, respectively. ^b^ Unspecified Cobb strain.

**Table 8:** Meta-regression of study factors on the overall pooled effect of replacing ITM with PTM (Bioplex Zn, Mn, Cu and Fe) at 11–100% dietary inclusion levels on the mineral excretion of broilers.

Study Factors	Cu Excretion (mg/kg)				Fe Excretion (mg/kg)				Mn Excretion (mg/kg)			
	Coefficient	SE	*p*-Value	*R*^2^ (%)	Coefficient	SE	*p*-Value	*R*^2^ (%)	Coefficient	SE	*p*-Value	*R*^2^ (%)
**Location**												
Africa	18.715	2.235	<0.001	29.0	94.977	4.667	<0.001	0.0	143.052	3.099	<0.001	58.0
Asia	-	-	-		-	-	-		−23.210	13.350	0.082	
North America	17.951	2.063	<0.001		−178.706	20.051	<0.001		19.007	3.295	<0.001	
Oceania	25.635	3.484	<0.001		14.771	10.478	0.159		132.412	4.824	<0.001	
South America	26.434	2.860	<0.001		94.404	4.429	<0.001		130.411	2.979	<0.001	
Europe	Referent ^a^				Referent ^a^				Referent ^a^			
**Year of study**	0.409	0.202	0.043	0.0	5.905	0.327	<0.001	0.0	0.403	0.220	0.067	3.0
**Breed/strain**												
Arbor Acres	0.2167	2.501	0.931	43.0	5.354	3.638	0.141	0.0	23.422	3.059	<0.001	43.0
Ross 308	−19.011	2.022	<0.001		−89.314	4.403	<0.001		−103.832	3.024	<0.001	
Ross 708	−9.163	1.929	<0.001		−290.492	20.557	<0.001		−114.957	3.016	<0.001	
Cobb ^b^	Referent ^a^				Referent ^a^				Referent ^a^			
Unspecified ^c^	−23.341	3.825	<0.001		-	-	-		-	-	-	
Cobb x Cobb	−2.957	2.967	0.319		21.224	74.474	0.776		-	-	-	
Cobb 500	-	-	-		-	-	-		−9.396	3.643	0.010	
**Age of birds**	−0.268	0.041	<0.001	24.0	−1.412	0.171	<0.001	0.0	−3.806	0.111	<0.001	4.0
**Number of birds**	−0.023	0.004	<0.001	53.0	−0.179	0.005	<0.001	40.0	−0.249	0.005	<0.001	51.0
**Study duration**	−0.261	0.034	<0.001	31.0	−1.665	0.122	<0.001	0.0	−3.021	0.084	<0.001	0.0
**Study factors**	**Zn (mg/kg)**											
	**Coefficient**	**SE**	***p*-value**	***R*^2^ (%)**								
**Location**												
Africa	88.365	2.579	<0.001	3.0								
Asia	-	-	-									
North America	25.874	2.608	<0.001									
Oceania	81.617	3.783	<0.001									
South America	80.776	2.566	<0.001									
Europe	Referent ^a^											
**Year of study**	0.623	0.152	<0.001	0.0								
**Breed/strain**												
Arbor Acres	16.528	2.282	<0.001	6.0								
Ross 308	−63.708	2.201	<0.001									
Ross 708	−58.499	2.172	<0.001									
Cobb ^b^	Referent ^a^											
Unspecified	-	-	-									
Cobb x Cobb	12.256	7.591	0.106									
Cobb 500	-	-	-									
**Age of birds**	−1.912	0.077	<0.001	0.0								
**Number of birds**	−0.127	0.003	<0.001	31.0								
**Study duration**	−1.569	0.055	<0.001	0.0								

SE: standard error; *R*^2^: proportion of between-study variance (heterogeneity) explained by the study factors. ^a^ Study location and broiler breed/strain were estimated by using Europe and Cobb as the baseline, respectively. ^b^ Unspecified Cobb strain. ^c^ Unspecified broiler breed.

**Table 9:** Meta-regression of study factors on the overall pooled effect of replacing ITM with lower inclusion rates (50–80%) PTM (Bioplex Zn, Mn, Cu and Fe) on the productive performance of broilers.

Study Factors	Total Feed Intake (kg/bird)				ADFI (g/day/bird)				BWG (g/bird)			
	Coefficient	SE	*p*-Value	*R*^2^ (%)	Coefficient	SE	*p*-Value	*R*^2^ (%)	Coefficient	SE	*p*-Value	*R*^2^ (%)
**Location**												
Africa	−0.066	0.027	0.014	0.0	−7.277	1.85	<0.001	9.0	60.165	6.905	<0.001	12.0
Asia	−0.058	0.024	0.016		−0.103	1.641	0.950		4.335	3.954	0.273	
North America	−0.054	0.026	0.036		−1.865	2.152	0.386		−11.113	7.076	0.116	
Oceania	−0.124	0.037	0.001		-	-	-		4.520	23.791	0.849	
South America	−0.057	0.024	0.017		-	-	-		−0.810	3.786	0.831	
Europe	Referent ^a^				Referent ^a^				Referent ^a^			
**Year of study**	−0.001	0.001	0.280	0.0	−0.097	0.148.0	0.512	0.0	0.153	0.281	0.586	0.0
**Breed/strain**												
Arbor Acres	-	-	-	0.0	−0.890	2.092	0.671	0.0	-	-	-	0.0
Ross 308	0.001	0.013	0.950		1.592	2.315	0.492		18.490	3.751	<0.001	
Ross 708	−0.005	0.071	0.941		-	-	-		-	-	-	
Cobb ^b^	−0.001	0.013	0.966		-	-	-		−0.526	3.720	0.888	
Vencobb	−0.006	0.018	0.737		-	-	-		−21.455	4.653	<0.001	
Hubbard JV	0.059	0.027	0.029		-	-	-		-	-	-	
Unspecified	−0.751	0.951	0.429		-	-	-		-	-	-	
Cobb 500	Referent ^a^				Referent ^a^				Referent ^a^			
**Age of birds**	<0.001	<0.001	0.789	0.0	−0.022	0.057	0.698	3.0	0.349	0.115	0.003	0.0
**Number of birds**	<0.001	<0.001	0.006	0.0	0.009	0.007	0.206	0.0	0.031	0.013	0.017	0.0
**Study duration**	<0.001	<0.001	0.817	0.0	−0.018	0.050	0.726	3.0	0.863	0.089	<0.001	0.0
**Study factors**	**FCR (g feed/g BWG)**				**ADG (g)**				**Mortality (%)**			
	**Coefficient**	**SE**	***p*-value**	***R*^2^ (%) **	**Coefficient**	**SE**	***p*-value**	***R*^2^ (%) **	**Coefficient**	**SE**	***p*-value**	***R*^2^ (%) **
**Location**												
Africa	−0.137	0.018	<0.001	0.0	11.263	1.710	<0.001	0.0	−1.000	3.015	0.740	32.0
Asia	−0.022	0.011	0.041		0.398	1.363	0.770		5.590	3.580	0.118	
North America	−0.031	0.018	0.086		-	-	-		0.765	3.888	0.844	
Oceania	−0.056	0.023	0.017		-	-	-		−1.500	5.114	0.769	
South America	−0.026	0.012	0.034		−0.061	1.717	0.972		0.47	3.184	0.883	
Europe	Referent ^a^				Referent ^a^				Referent ^a^			
**Year of study**	−0.0004	0.001	0.677	0.0	−0.141	0.109	0.195	0.0	0.056	0.251	0.822	0.0
**Breed/strain**												
Arbor Acres	0.020	0.020	0.314	0.0	3.469	1.494	0.020	0.0	0.651	2.072	0.753	28.0
Ross 308	0.017	0.019	0.378		0.074	1.717	0.966		−0.419	2.174	0.847	
Ross 708	0.005	0.034	0.880		-	-	-		0.536	3.212	0.868	
Cobb ^b^	0.016	0.020	0.420		-	-	-		-	-	-	
Vencobb	0.031	0.024	0.190		-	-	-		-	-	-	
Hubbard JV	0.031	0.033	0.341		-	-	-		-	-	-	
Unspecified	-	-	-		-	-	-		-	-	-	
Cobb 500	Referent ^a^				Referent ^a^				Referent ^a^			
**Age of birds**	<0.001	<0.001	0.981	0.0	0.166	0.039	<0.001	15.0	−0.179	0.084	0.032	24.0
**Number of birds**	<0.001	<0.001	0.442	0.0	−0.008	0.006	0.179	0.0	−0.004	0.011	0.712	34.0
**Study duration**	<0.001	<0.001	0.230	0.0	0.140	0.034	<0.001	10.0	−0.179	0.084	0.032	24.0

SE: standard error; *R*^2^: proportion of between-study variance (heterogeneity) explained by the study factors. ^a^ Study location and broiler breed/strain were estimated by using Europe and Cobb 500 as the baseline, respectively. Mortality was the only exception which used Cobb as a referent in this meta regression. ^b^ Unspecified Cobb strain.

**Table 10:** Effect of supplementing PTM (Bioplex Zn, Mn, Cu and Fe) at 50–80% of ITM levels in diets containing two different levels of SBM on the carbon footprint of broiler production.

	Low-SBM Diet			High-SBM Diet		
Category/Functional Unit	Baseline	PTM	% Change	Baseline	PTM	% Change
Total overall gross emissions (t CO_2_-eq)	482.0	465.2	−3.49%	515.4	497.3	−3.51%
**Feed emission intensity**						
Emissions per bird (kg CO_2_-eq/bird)	4.11	3.92	−4.07%	4.45	4.25	−4.10%
Emissions per live weight (kg CO_2_-eq/kg LW)	1.64	1.57	−4.07%	1.78	1.70	−4.10%
**Total emission intensity**						
Emissions per bird (kg CO_2_-eq/bird)	5.08	4.88	−4.50%	5.44	5.21	−4.50%
Emissions per live weight (kg CO_2_-eq/kg LW)	2.03	1.95	−4.50%	2.17	2.09	−4.50%

SBM: soybean meal; PTM: proteinate trace minerals; LW: live weight.

## Data Availability

All data and related tables generated during this meta-analysis are included in the published review and its Supplementary Information files. The review was not registered previously. The review protocol followed previous meta-analysis publications and was not prepared for external accession.

## Supplementary Materials

The following supporting information can be downloaded at: https://www.mdpi.com/article/10.3390/ani15213062/s1, Figure S1: Forest plot of the effect of proteinate trace mineral supplementation on Total Feed Intake (kg/bird) of broilers; Figure S2: Forest plot of the effect of proteinate trace mineral supplementation on Average Daily Feed Intake (g/day/bird) of broilers; Figure S3: Forest plot of the effect of proteinate trace mineral supplementation on Average Daily Gain (g) of broilers; Figure S4: Forest plot of the effect of proteinate trace mineral supplementation on Body Weight Gain (g/bird) of broilers; Figure S5: Forest plot of the effect of proteinate trace mineral supplementation on Final Body Weight (g/bird) of broilers; Figure S6: Forest plot of the effect of proteinate trace mineral supplementation on Feed Conversion Ratio (g feed/g BWG) of broilers; Figure S7: Forest plot of the effect of proteinate trace mineral supplementation on Mortality (%) of broilers; Figure S8: Forest plot of the effect of proteinate trace mineral supplementation on Cu excretion levels (mg/kg) of broilers; Figure S9: Forest plot of the effect of proteinate trace mineral supplementation on Fe excretion levels (mg/kg) of broilers; Figure S10: Forest plot of the effect of proteinate trace mineral supplementation on Mn excretion levels (mg/kg) of broilers; Figure S11: Forest plot of the effect of proteinate trace mineral supplementation on Zn excretion levels (mg/kg) of broilers; Table S1: Ingredient, nutrient composition and emission intensity of formulated broiler diets used in the life cycle assessment; Table S2: Data describing broiler performance and production characteristics in the baseline and proteinate trace mineral (PTM) scenarios; Table S3: Data quality indicators describing activity and inventory data in the modelled LCA scenarios; Table S4: Breakdown of greenhouse gas emissions output from the life cycle assessment of baseline and proteinate trace mineral (PTM) scenarios managed on low- and high-soya bean meal (SBM) diets.

## Abbreviations

ADG—Average Daily Gain; ADFI—Average Daily Feed Intake; AHDB—Agriculture and Horticulture Development Board; BW—Body weight; BWG—Body Weight Gain; CFP—Carbon Footprint; CMA—Comprehensive Meta-Analysis; Cu—Copper; CI—Confidence Interval; DEFRA—Department for Environment, Food and Rural Affairs; FCR—Feed Conversion Ratio; Fe—Iron; FI—Feed Intake; GHG—Greenhouse Gas; GWP—Global Warming Potential; IPCC—Intergovernmental Panel on Climate Change; ITM—Inorganic Trace Minerals; LCA—Life Cycle Assessment; MD—Mean difference; Mn—Manganese; PTM—Proteinate Trace minerals; PRISMA—Preferred Reporting Items for Systematic Reviews and Meta-Analyses; RBV—Relative Bioavailability; RMD—Raw mean difference; ROI—Return On Investment; SBM—Soybean Meal; SD—Standard deviation; Zn—Zinc.
