# Supplementary material for: The Effect of Chelated Trace Mineral Supplementation in the Form of Proteinates on Broiler Performance Parameters and Mineral Excretion: A Meta-Analysis
Source: Animals (Basel). 2025 Oct 22;15(21):3062. doi: 10.3390/ani15213062 (PMC12607354; doi:10.3390/ani15213062)
Supplement: Supplementary file 1 [file animals-15-03062-s001.zip › Final Supplementary files/Supplementary data files Forest Plots for All Data and Mineral Excretion.docx]

**SUPPLEMENTARY FIGURES S1-S11**

**Note on supplementary Figures S1–S11:** Forest plots of the effect of proteinate trace mineral supplementation on specified production performance parameters and mineral excretion data for broilers. Study name refers to the reference of that study. Raw mean differences (difference in means) represent the effect size estimate. Each square represents the mean effect size for that study. Thus, the squares to the right of the zero mid-line represent an increase in feed intake, squares to the left of the 0 mid-line indicate a decrease in feed intake and squares at the 0 mid-line indicate no effect on feed intake. The upper and lower limit of the line connected to the square represents the upper and lower 95% confidence interval (CI) for the effect size. The size of the square reflects the relative weighting of the study to the overall effect size estimate, with larger squares representing greater weight. The diamond at the bottom represents the overall effect size estimate.

**Figure S1:** Forest plot of the effect of proteinate trace mineral supplementation on Total Feed Intake (kg/bird) of broilers

**Figure S2:** Forest plot of the effect of proteinate trace mineral supplementation on Average Daily Feed Intake (g/day/bird) of broilers

**Figure S3:** Forest plot of the effect of proteinate trace mineral supplementation on Average Daily Gain (g) of broilers

**Figure S4:** Forest plot of the effect of proteinate trace mineral supplementation on Body Weight Gain (g/bird) of broilers

**Figure S5:** Forest plot of the effect of proteinate trace mineral supplementation on Final Body Weight (g/bird) of broilers

**Figure S6:** Forest plot of the effect of proteinate trace mineral supplementation on Feed Conversion Ratio (g feed/g BWG) of broilers

**Figure S7:** Forest plot of the effect of proteinate trace mineral supplementation on Mortality (%) of broilers

**Figure S8:** Forest plot of the effect of proteinate trace mineral supplementation on Cu excretion levels (mg/kg) of broilers

**Figure S9:** Forest plot of the effect of proteinate trace mineral supplementation on Fe excretion levels (mg/kg) of broilers

**Figure S10:** Forest plot of the effect of proteinate trace mineral supplementation on Mn excretion levels (mg/kg) of broilers

**Figure S11:** Forest plot of the effect of proteinate trace mineral supplementation on Zn excretion levels (mg/kg) of broilers
