# Supplementary material for: The Effect of Chelated Trace Mineral Supplementation in the Form of Proteinates on Broiler Performance Parameters and Mineral Excretion: A Meta-Analysis
Source: Animals (Basel). 2025 Oct 22;15(21):3062. doi: 10.3390/ani15213062 (PMC12607354; doi:10.3390/ani15213062)
Supplement: Supplementary file 1 [file animals-15-03062-s001.zip › Final Supplementary files/Supplementary tables for LCA data revised Oct2025.docx]

**SUPPLEMENTARY MATERIAL**

| **Table S1.** Ingredient, nutrient composition and emission intensity of formulated broiler diets used in the life cycle assessment | | | | | | | | | |
| --- | --- | --- | --- | --- | --- | --- | --- | --- | --- |
|  | Starter phase (day 0 - 10) | |  | Grower phase (day 11 - 24) | |  | Finisher phase (day 25 - slaughter) | |  |
| Ingredient (%) | High-SBM | Low-SBM |  | High-SBM | Low-SBM |  | High-SBM | Low-SBM |  |
| Wheat | 54.42 | 54.97 |  | 55.20 | 54.30 |  | 56.02 | 51.74 |  |
| Whole wheat | - |  |  | 5.00 | 5.00 |  | 10.00 | 10.00 |  |
| Rapeseed whole | 5.00 | 5.00 |  | 7.50 | 7.50 |  | 10.00 | 10.00 |  |
| Soybean meal | 33.50 | 30.00 |  | 25.50 | 22.00 |  | 18.00 | 16.00 |  |
| Sunflower meal | - | 2.50 |  | - | 3.75 |  | - | 5.00 |  |
| Vegetable oil blend | 0.70 | 0.50 |  | 2.00 | 2.40 |  | 2.50 | 3.50 |  |
| Soya oil | 2.70 | 3.20 |  | 1.60 | 1.80 |  | 0.70 | 1.00 |  |
| Limestone | 1.40 | 1.40 |  | 1.20 | 1.20 |  | 1.00 | 1.00 |  |
| Monocalcium phosphate | 0.92 | 0.85 |  | 0.75 | 0.65 |  | 0.50 | 0.45 |  |
| Sodium bicarbonate | 0.18 | 0.18 |  | 0.15 | 0.15 |  | 0.18 | 0.18 |  |
| Sodium chloride | 0.20 | 0.20 |  | 0.17 | 0.17 |  | 0.15 | 0.15 |  |
| Lysine-HCl | 0.28 | 0.38 |  | 0.25 | 0.33 |  | 0.31 | 0.34 |  |
| DL-methionine | 0.32 | 0.35 |  | 0.30 | 0.32 |  | 0.26 | 0.25 |  |
| L-threonine | 0.10 | 0.15 |  | 0.10 | 0.12 |  | 0.10 | 0.11 |  |
| Valine | - | 0.04 |  |  | 0.03 |  | - | - |  |
| Enzyme | 0.03 | 0.03 |  | 0.03 | 0.03 |  | 0.03 | 0.03 |  |
| Premix | 0.25 | 0.25 |  | 0.25 | 0.25 |  | 0.25 | 0.25 |  |
| Total | 100 | 100 |  | 100 | 100 |  | 100 | 100 |  |
| **Nutrient composition** | | | | | | | | | |
| Energy (MJ/kg) | 12.7 | 12.7 |  | 13.1 | 13.1 |  | 13.4 | 13.4 |  |
| Protein (%) | 22.8 | 22.2 |  | 20.0 | 19.6 |  | 18.5 | 17.8 |  |
| **Embedded emissions in ration production** |  |  |  |  |  |  |  |  |  |
| Emission Intensity (kgCO_2_e tonne^-1^) | 1,247 | 1,245 |  | 1,040 | 1,011 |  | 992 | 862 |  |
| Low- and high-SBM diet formulations employed are adopted from those presented in Salami et al. [99] | | | | | | | | |  |

**Table S2.** Data describing broiler performance and production characteristics and LCA input variables in the baseline and proteinate trace mineral (PTM) scenarios

| **Item** | **ITM Baseline** | **PTM Scenario** |
| --- | --- | --- |
| Number of birds placed (n) | 100,000 | 100,000 |
| Mortality (%) | 5.20 | 4.62 |
| Average daily feed intake (g/d) | 93.3 | 92.3 |
| Average daily gain (g/d) | 54.9 | 56.9 |
| Slaughter weight (kg) | 2.5 | 2.5 |
| Number of days to slaughter (d) | 44 | 43 |
| Total feed - starter (kg) | 65,073 | 62,523 |
| Total feed - grower (kg) | 114,361 | 109,880 |
| Total feed - finisher (kg) | 223,337 | 214,586 |
| Electricity (kWh) | 120,782 | 120,782 |
| Diesel (l) | 127 | 127 |
| Disinfectant (l) | 2,028 | 2,028 |
| Mains water (m^3^) | 2,704 | 2,704 |
| Total birds finished (n) | 94,800 | 95,377 |
| Total liveweight finished (kg) | 237,000 | 238,442 |
| Kill-out (%) | 70.5 | 70.5 |

**Table S3.** Data Quality Indicators describing activity and inventory data in the modelled LCA scenarios

| Category | Description / source | Precision | Completeness | Temporal | Technological | Geographical |
| --- | --- | --- | --- | --- | --- | --- |
| **Activity Data** |  |  |  |  |  |  |
| Performance and production | Daily feed intake, daily weight gain, mortality: meta-analysis in present study | Good | Good | Medium | Good | Medium |
| Farm general consumption | Electricity, fuel, bedding, disinfectant, water: averages derived from primary data collected in 168 commercial European broiler farm assessments 2023-2025 (Alltech E-CO_2_) | Good | Good | Good | Good | Good |
| Feed Rations | Formulations and assumptions adopted from recent published study by Salami et al. [93], based on diets originally derived from Leinonen et al. [18] | Good | Medium | Good | Good | Medium |
| **Inventory Emission Factor Data** |  |  |  |  |  |  |
| Placed birds | Embedded emissions in day-old chicks: average derived from results of 19 commercial European rearer-breeder-hatchery assessments 2021-2025 (Alltech E-CO_2_) | Good | Good | Good | Good | Good |
| General consumption | Electricity, fuel, bedding, disinfectant, water source: DEFRA [104] | Good | Good | Good | Good | Good |
| Transport | Vehicle type-specific, source: DEFRA [104] | Good | Good | Good | Good | Good |
| Feed | Individual ingredients, based on European market mix, source: FeedPrint database [103] | Good | Good | Good | Medium | Good |
| Impact assessment calculations | Equations and coefficients pertinent to estimating N_2_O and CH_4_ from manure deposition and management at tier 2 level, source IPCC [102] | Good | Good | Medium | Good | Medium |
| Exported litter | Nutrient content of exported litter values, source: Nutrient Management Guide RB209 [105] | Good | Medium | Good | Medium | Good |
| Inorganic fertiliser production | Nitrogen fertiliser, region specific: Fertilizers Europe [106] | Good | Good | Medium | Good | Good |

Data Quality Indicator (DQI) approach derived from Carbon Trust (2022), where: Precision = the source of activity or emissions data; Completeness = how completely does the data represent the intended emission boundary; Temporal = the age of the data, relative to the inventory year; Technological = representation of the core activity used in the process modelled; Geographic = the region the data represent; and indicators are on a three-level range consisting of ‘good’, medium’ and ‘poor’.

**Table S4.** Breakdown of greenhouse gas emissions output from the life cycle assessment of baseline and proteinate trace mineral (PTM) scenarios managed on low- and high-soya bean meal (SBM) diets

|  | **ITM Baseline** | |  | **PTM Scenario** | |
| --- | --- | --- | --- | --- | --- |
| Emissions Contributing Category  (kg CO_2_-eq) | Low-SBM | High-SBM |  | Low-SBM | High-SBM |
| Placed day-old chicks | 34,694 | 34,694 |  | 34,694 | 34,694 |
| Litter/bedding | 8,306 | 8,306 |  | 8,306 | 8,306 |
| Water use | 479 | 479 |  | 479 | 479 |
| Disinfectants | 2,150 | 2,150 |  | 2,150 | 2,150 |
| Starter feed | 80,985 | 81,133 |  | 77,812 | 77,954 |
| Grower feed | 115,673 | 118,922 |  | 111,141 | 114,263 |
| Finisher feed | 192,512 | 221,464 |  | 184,970 | 212,787 |
| Transport delivery of feed | 4,850 | 4,850 |  | 4,660 | 4,660 |
| Electricity | 27,175 | 27,175 |  | 27,175 | 27,175 |
| Fossil fuel | 417 | 417 |  | 417 | 417 |
| Manure management | 14,978 | 16,006 |  | 13,620 | 14,607 |
| Deadstock disposal | 78 | 78 |  | 70 | 70 |
| Exported manure | -296 | -296 |  | -296 | -296 |
| **Total emissions** | **482,002** | **515,377** |  | **465,197** | **497,265** |
